# ROS-Mediated Decline in Maximum Ca^2+^-Activated Force in Rat Skeletal Muscle Fibers following *In Vitro* and *In Vivo* Stimulation

**DOI:** 10.1371/journal.pone.0035226

**Published:** 2012-05-22

**Authors:** Travis L. Dutka, Esther Verburg, Noni Larkins, Kristin H. Hortemo, Per K. Lunde, Ole M. Sejersted, Graham D. Lamb

**Affiliations:** 1 Department of Zoology, La Trobe University, Melbourne, Australia; 2 Institute for Experimental Medical Research, University of Oslo and Oslo University Hospital, Oslo, Norway; University of Queensland, Australia

## Abstract

We hypothesised that normal skeletal muscle stimulated intensely either *in vitro* or *in situ* would exhibit reactive oxygen species (ROS)-mediated contractile apparatus changes common to many pathophysiological conditions. Isolated soleus (SOL) and extensor digitorum longus (EDL) muscles of the rat were bubbled with 95% O_2_ and stimulated *in vitro* at 31°C to give isometric tetani (50 Hz for 0.5 s every 2 s) until maximum force declined to ≤30%. Skinned superficial slow-twitch fibers from the SOL muscles displayed a large reduction (∼41%) in maximum Ca^2+^-activated specific force (F_max_), with Ca^2+^-sensitivity unchanged. Fibers from EDL muscles were less affected. The decrease in F_max_ in SOL fibers was evidently due to oxidation effects on cysteine residues because it was reversed if the reducing agent DTT was applied prior to activating the fiber. The GSH∶GSSG ratio was ∼3-fold lower in the cytoplasm of superficial fibers from stimulated muscle compared to control, confirming increased oxidant levels. The presence of Tempol and L-NAME during *in vitro* stimulation prevented reduction in F_max_. Skinned fibers from SOL muscles stimulated *in vivo* at 37°C with intact blood supply also displayed reduction in F_max_, though to a much smaller extent (∼12%). Thus, fibers from muscles stimulated even with putatively adequate O_2_ supply display a reversible oxidation-induced decrease in F_max_ without change in Ca^2+^-sensitivity, consistent with action of peroxynitrite (or possibly superoxide) on cysteine residues of the contractile apparatus. Significantly, the changes closely resemble the contractile deficits observed in a range of pathophysiological conditions. These findings highlight how readily muscle experiences ROS-related deficits, and also point to potential difficulties when defining muscle performance and fatigue.

## Introduction

Reactive oxygen and nitrogen species (ROS and RNS) are thought to have a major role in the skeletal muscle weakness observed in a host of pathophysiological conditions such as sepsis [1], [2], rheumatoid arthritis and other inflammatory conditions [3], [4], and heart failure and stroke [5], [6], [7], [8]. In many of these conditions the muscle weakness is associated with a reduction in maximum specific force (F_max_) independent of muscle atrophy, and occurs without any change in Ca^2+^-sensitivity [9]. However, it is not known which specific oxidant(s) causes the dysfunction, which protein sites are involved, whether the dysfunction is acutely reversible, and whether different fiber types are affected to the same degree. It is also unclear whether or how readily normal skeletal muscle displays the same specific dysfunction in conditions where ROS and RNS levels are elevated.

Application of exogenous ROS and RNS have been shown to have various effects on maximum force production and/or Ca^2+^ sensitivity of the myofilaments [10], [11], [12], [13], [14], [15], [16], [17], [18], [19], as well as on the activity of the sarcoplasmic reticulum (SR) Ca^2+^ pumps (SERCA) [20], and the SR Ca^2+^ release channels (ryanodine receptors) [21], [22] (for review see [23], [24], [25]). ROS and RNS are generated in muscle with both hypoxia and hyperoxia [26], [27], as well as with elevated muscle temperature *in vitro* [28], [29]. Interestingly, it has been found that a 30 min period of anoxia reduced F_max_ in rat soleus (SOL) muscle bundles by ∼32% without change in Ca^2+^-sensitivity, in association with increased nitrotyrosine levels, and also that the effects could be prevented with the nitric oxide synthase inhibitor L-NMMA [30]. It was concluded that the contractile changes likely resulted from the effects of increased levels of peroxynitrite. Furthermore, when mouse EDL muscles bubbled *in vitro* with 95% O_2_ were maintained at 37°C for 30 min, F_max_ also decreased substantially (∼50%) without change in Ca^2+^-sensitivity, with effects prevented by the superoxide dismutase mimetic Tempol [28]. The decrease in F_max_ was suggested to be due to increased superoxide, but might also be due to effects of peroxynitrite, which is readily generated when superoxide and nitric oxide levels are elevated [31], [32], [33], [34]. In apparent accord, application of peroxynitrite causes a reduction in F_max_ with little or no change in Ca^2+^-sensitivity in both limb muscles and diaphragm [18], [35], interestingly with greater effect in slow-twitch SOL muscle fibers than in fast-twitch EDL fibers [18]. Application of superoxide also causes a decrease in F_max_ in diaphragm fibers, though its effects on Ca^2+^-sensitivity were not ascertained [11]. In contrast, related ROS and RNS, including H_2_O_2_, hydroxyl, NO and GSNO, have quite different actions on limb muscle fibers, changing Ca^2+^-sensitivity more readily than F_max_ or having little if any effect (as in case of H_2_O_2_) [12], [13], [17], [19].

Importantly, ROS and RNS are known to be generated in muscle with activity [23], [24], [36]. Various studies of muscle function have utilized *in vitro* stimulation of whole muscles or muscle bundles with 95% O_2_ at temperatures ≥30°C. The high O_2_ levels are used to avoid hypoxia in deep regions of the muscle [37], but the combination of stimulation and hyperoxia seem assured to lead to elevated levels of ROS and RNS, raising the question of whether resulting ROS-related effects influence the muscle properties found. An important related question and comparison is what effects, if any, occur in muscles stimulated *in situ* with their blood supply intact.

This study investigates whether the increased levels of ROS/RNS generated by stimulating an *in vitro* whole muscle preparation in typical oxygenation conditions, causes dysfunction of the contractile proteins in superficial, well-oxygenated fibers, and compares results found in an *in situ* muscle preparation with more physiological oxygenation and perfusion. We hypothesised that normal healthy muscle can be made to exhibit the hallmarks of ROS-mediated contractile apparatus changes characteristic of many pathophysiological conditions if the muscles experience abnormal O_2_ supply and perfusion. We examined maximum force production and Ca^2+^-sensitivity in skinned fibers from muscle stimulated *in vitro* and *in situ*, comparing each to matching fibers from the non-stimulated contralateral muscles, and further tested the effects of particular treatments to counter or reverse possible oxidation effects. By examining contractile properties of skinned fibers under standardized conditions mimicking the resting cytoplasmic environment, it was possible to determine whether there were any changes in fiber characteristics without confounding effects arising from the alterations in cytoplasmic conditions that occur with repeated stimulation.

## Methods

### Ethics Statement

All animal *in vitro* experiments were performed at La Trobe University (Melbourne Campus, Australia) with approval of the La Trobe University Animal Ethics Committee (animal ethics number AEC06-09-L). All animal *in vivo* experiments were conducted at the University of Oslo (Norway) in accordance with current regulations and approved by the Norwegian Animal Research Authority, approval ID 2310.

### In vitro whole muscle preparation and experimental protocol

Seventeen male Long-Evans hooded rats and one male Wistar rat, 3–8 mo old, were kept at the La Trobe University Central Animal House in cages in a climate-controlled room with 12/12 h light/dark cycle and food and water given *ad libidum*. The rats were killed by overdose of isoflurane and their soleus ‘SOL’ or extensor digitorum longus ‘EDL’ muscles dissected out and attached between a glass hook and an insulated force-transducer in an *in vitro* bath filled with Krebs-Ringer solution at 31°C. The solution was bubbled with 95% O_2_ and 5% CO_2_, with final pH of ∼7.4. Force responses were sampled at 1000 Hz on a personal computer (Powerlab hardware and Chart 5 software, ADInstruments Australia). Following 10 min of equilibration, the muscle was electrically stimulated with field stimulation to contract isometrically. Muscle length and stimulation voltage were varied to give maximum twitch force and voltage set at >1.5 times this level, and then the force frequency behaviour examined with ∼5 tetani evoked at various frequencies (10–100 Hz). After a further 5 min rest the stimulation protocol was started. In one set of experiments one of each contralateral pair of SOL muscles was subjected to a prolonged train of tetani, each elicited with 1 ms pulses at 50 Hz for 0.5 s every 2 s, until the peak tetanic force dropped to less than 30% of its initial level (on average muscles were stimulated for ∼5 min). In most instances, following two minutes rest the muscle was subjected to: a) one further 0.5 s 50 Hz stimuli in order to test whether there was any rapid recovery of the tetanic force, b) another such stimulus at higher voltage to verify that the stimulating voltage was still supramaximal, and c) finally tested with a single prolonged 50 Hz stimulus until the force plateau was reached in order to assess maximum tetanic force. Immediately afterwards the muscle was removed from the *in vitro* bath and pinned at resting length in a silica-gel lined Petri dish filled with paraffin oil. The dish was then put on ice in order to maintain the muscle temperature at ∼6–8°C. Contralateral muscles were placed directly into paraffin oil in a Petri dish and put on ice, and served as the rested control muscle. In another set of experiments, both SOL muscles from a given rat were stimulated *in vitro* as above, but one was left to recover in oxygenated Krebs-Ringer solution for 45 min following stimulation before being put in oil in the Petri dish and on ice.

### In vivo whole muscle preparation and experimental protocol

Five male Wistar rats, 3–4 months old (supplied by Taconic, Skensvedt Denmark) were kept for at least 1 wk at the animal facility at Oslo University Hospital Ullevaal in cages in a climate-controlled room with 12/12 h light/dark cycle and food and water given *ad libidum*. Each rat was anesthetised in a chamber filled with 1∶3 O_2_/N_2_O with 4% isoflurane (Abbott no. 506949, Chicago, Ill. USA) and then the rat was intubated and attached to a respirator (Zoovent, Triumph Technical Services LTD, London, UK) and kept under anesthesia with 1∶3 O_2_/N_2_O gas mixture with 2–3% isoflurane for the remainder of the experiment. Blood pressure was measured with a microtip pressure catheter (SPR-407, Millar Instruments Inc, Houston TX, USA) inserted via the right arteria carotis communis and led retrograde to the aortic arch, and used to monitor anesthesia depth and general condition of the animal. The animal was kept on a heated table (37°C) during the experiment.

The right SOL muscle was carefully dissected free from the surrounding tissues, leaving the muscles blood supply intact. The calcaneus was cut and tendons to the other calf muscles separated and cut, leaving just the SOL tendon. This tendon was attached to the lever of a servo-controlled force transducer (model 305B Aurora Scientific, Canada). The tibia was clamped immobile. The muscle was kept moist and at 37°C by dripping warmed 0.9% NaCl solution onto the muscle. The sciatic nerve was cut to prevent retrograde transmission of the stimulation current. The SOL muscle was activated to contract isometrically by direct stimulation with platinum electrodes with 1 ms pulses at 8 V (pulsar 6 bp, FHC Brunswick, ME, USA), at the proximal and distal end of the muscle. Optimum length and voltage was set using twitch force, followed by a few test tetani at various frequencies (10–100 Hz). After 5 min rest, the stimulation protocol was started, consisting of the same repeated 50 Hz tetani protocol as the *in vitro* experiments (0.5 s every 2 s, 1 ms pulses), with the only difference being that the protocol was continued for 10 min in every case. Force, aortic pressure, muscle surface temperature and stimulation pulses were sampled at 2000 Hz (National Instruments hardware and Labview software). Immediately after the stimulation protocol, the muscle was removed and pinned under paraffin oil in a Petri dish, which was put on ice. The contralateral muscle was dissected out and also pinned in the Petri dish, and served as resting control. At the end of the experiment the rat was sacrificed by decapitation.

### Single skinned fiber preparation

Skinned fibers were prepared from SOL and EDL muscles as described previously [12], [38]. Using the muscles kept under paraffin oil on ice, single fibers were dissected from the superficial region of the muscle (<7 fibers deep), and mechanically skinned using a pair of forceps to peel back the surface membrane. These skinned fiber segments (∼2 mm long) were then attached between forceps and a force transducer (Memscap AE801, Skoppum, Norway). The fiber segment was stretched to 120% of slack length (the length at which the fiber just starts to produce passive force), and the diameter of the fiber segment measured under high magnification at two or three representative points along its length. The fiber segment was then transferred to the relaxing buffer solution (see solutions section).

### Solutions

The Krebs-Ringer solution for the *in vitro* experiments contained (in mM): 122 NaCl, 2.8 KCl, 1.2 MgSO_4_, 1.2 KH_2_PO_4_, 25 NaHCO_3_, with 1.3 CaCl_2_ and 5 D-Glucose added on the day of the experiment. The solution was bubbled with 95% O_2_ and 5% CO_2_ at 31°C for at least 30 min prior to the start of the stimulation in order to ensure stable oxygen tension, temperature and pH (7.4). The solutions for the skinned fibers were the same as previously described [12], [38]. The relaxing solution contained (in mM) 50 EGTA, 90 Hepes, 10.3 total Mg^2+^ (giving 1 mM free), 126 K^+^, 36 Na^+^, 8 ATP and 10 creatine phosphate (CP), pH 7.10, pCa ( = −log_10_[Ca^2+^])>9. Maximal Ca^2+^-activating solution contained (in mM) 50 CaEGTA, 9, Hepes, 8.1 total Mg^2+^ (giving 1 mM free), 126 K^+^, 36 Na^+^, 8 total ATP, 10 CP, pH 7.10 and pCa 4.7. Solutions with free [Ca^2+^] heavily buffered at intermediate levels (pCa 6.7 to 4.7) were obtained by mixing appropriate volumes of the relaxing solution and maximal activating solutions. The strontium (Sr) activating solution at pSr 5.3 was made by mixing relaxing buffer with maximal Sr activating solution containing (mM): 40 SrEGTA, 10 EGTA, 90 Hepes, 8.5 Mg^2+^ (giving 1 mM free), 126 K^+^, 36 Na^+^, 8 ATP, 10 CP, pH 7.10, pSr 3.7. Where required, 10 mM dithiodithreitol (DTT) was added to relaxing solution from a 1 M stock prepared in distilled water. Tempol (4-Hydroxy-2, 2, 6, 6-tetramethylpiperidine), a superoxide dismutase mimetic, and L-NAME (N-Nitro-L-arginine methyl ester hydrochloride), a nitric oxide (NO^•^) scavenger, were directly dissolved in the Krebs-Ringer solution at a concentration of 1 and 3 mM respectively.

### Measurement of force-[Ca^2+^] relationship in skinned fibers

Using skinned fibers the force-[Ca^2+^] relationship of the contractile proteins was assessed by exposing the fibers to a sequence of solutions with the free [Ca^2+^] very strongly buffered at progressively higher levels over the relevant range (pCa 6.7 to 4.7), with other conditions mimicking the normal cytoplasm with respect to osmolality, ionic strength, [K^+^], pH and free [Mg^2+^] [12], [38]. All measurements on skinned fibers were performed at room temperature (22–24°C). Individual fibers segments were transferred into a given solution until force stabilized (∼5–10 s) before moving the fiber segment to the next solution. Where required, a fiber was bathed for 10 min in relaxing solution containing 10 mM DTT either before any activation sequences or after two such sequences, as appropriate. Each fiber was also assessed as being fast or slow type according to its response to Sr^2+^ activation at pSr 5.3, which elicits little or no force in fast type fibers and near-maximal force in slow type fibers [38], [39], [40]. In the case of SOL muscle, only fibers classified as slow type were included in the present study, this typically being >85% of the total fibers dissected from the SOL muscles. Force was sampled at 1000 Hz using Chart 5 and AD Instruments or Labview and National Instruments CompaqDAQ). Maximum Ca^2+^ activated specific force (F_max_) was normalized to fiber cross-sectional area; the fiber cross-sectional area calculation was made assuming either a circular profile if the diameter was similar at all measured points along the fiber segment, or an ellipsoidal profile with dimensions corresponding to the largest and smallest diameter measurements. The two fibers with a calculated maximum Ca^2+^ activated force (F_max_) greater than 500 mN/mm^2^ were omitted from the analysis, as such high values are not possible and are most likely the result of inaccurate measurement of diameter due to non-ellipsoidal fiber-cross-section (one STIM fiber and one CC fiber). The force-pCa relationship in each fiber was determined by normalizing the force measured at each pCa to the maximum force reached in that sequence, and then fitting a Hill curve to each individual case for every individual fiber in order to calculate the half-maximal activating [Ca^2+^] (pCa_50_) and the Hill coefficient (*h*). Mean values of these parameters were then calculated across fibers and across treatments as appropriate (see Tables 1 & 2), and statistical tests based on these numbers. For ease of visualization of mean data, fits to the mean force-pCa data rather than the full array of individual fits are shown. However, caution should be used when viewing the fit to the mean data because even though it will always give a reliable indication of the mean pCa_50_ value, it will inevitably give a shallower slope (smaller Hill coefficient) than actually pertained in the individual cases whenever there is any spread in position (i.e. pCa_50_ values) of the individual curves.

**Table 1:** Ca^2+^-sensitivity of contractile apparatus in *in vitro* fibers.

*SOL*	*CC*	*STIM*	*STIM*	*STIM+REC*	*CC*	*STIM*	*STIM+DTT*
**pCa_50_**	5.96±0.02	5.94±0.02	5.94±0.04	5.98±0.03	6.02±0.02	6.02±0.02	6.01±0.08
***h***	3.7±0.3	3.2±0.2	5.6±0.7	4.5±0.6	3.5±0.4	3.0±0.2	3.4±0.4
***n***	24	18	9	9	7	6	6
***EDL***							
**pCa_50_**	5.85±0.02	5.82±0.02					
***h***	5.8±0.8	6.3±0.9					
***n***	12	11					

Hill curves fit to force-pCa data for each individual fiber for *in vitro* data in Figures 2 & 3; values are means (± SE) of pCa_50_ and Hill coefficient *h* averaged across all fibers (n) from muscles given indicated treatment. Data set comparing CC with STIM and STIM+DTT fibers (2 rats) are a subset of fibers shown in left-hand columns comparing CC with STIM (7 rats), whereas data set comparing STIM with STIM+REC fibers are from a separate set of experiments (2 rats). No significant differences were found.

**Table 2:** Ca^2+^-sensitivity of contractile apparatus in *in vivo* fibers.

	*CC*	*STIM*	*CC*	*STIM*	*STIM+DTT*
**pCa_50_**	6.10±0.01	6.11±0.04	6.08±0.01	6.16±0.05	6.17±0.05
***h***	2.6±0.2	2.3±0.1	3.1±0.2	2.3±0.2	2.7±0.4
***n***	13	12	7	7	5

Hill curve fit to force-pCa data for each individual fiber from *in vivo* stimulation experiments, data presented as in Table 1. Data set comparing CC with STIM and STIM+DTT fibers (from 3 rats) are a subset of experiments comparing CC with STIM fibers (5 rats). No significant differences were found.

### Determination of GSH: GSSG ratio in superficial bundles

Bundles of superficial fibers were dissected from around the periphery of three stimulated muscles and the contralateral non-stimulated muscles and prepared for GSSG and total GSH measurements as per the manufacturer's instructions (Cayman Glutanione assay Catalog No. 703002). Briefly, fiber bundles were blotted on filter paper to remove any blood and immediately homogenised 1∶10 (mass∶volume) in cold MES buffer (as supplied by manufacturer, Cayman Glutathione assay) and homogenized. The mixture was then centrifuged at 10,000 g for 15 min at 4°C and the supernatant collected. Samples were deproteinated using MPA regent (1.25 M metaphosphoric acid) in equal volume to the supernatant and immediately vortexed. Samples were left for 5 min at room temperature and then centrifuged at 2,000 g for 2 min. The supernatant was removed and stored at −20°C until analysed. On the day of the assay 50 µl/ml TEAM reagent (4 M triethanolamine) was added to each sample and mixed thoroughly. In order to quantify GSSG exclusive of GSH, 10 µl of 2-vinylpyridine (1 M 2-vinylpyridine) was added per ml of GSSG specific sample. At this point all samples were further diluted with MES buffer. Each plate was loaded with duplicate standard curves for both GSSG and GSH, along with triplicates of each sample. Each well was loaded with the assay cocktail (in accordance with manufacturer's instructions) and incubated in the dark on an orbital shaker for 2 min. The absorbance of each well was measured at 5 min intervals for 30 min at 405–414 nm using RA Anthos Reader 2010 (Biochrom, Cambridge UK) and ADAP Plus software (Biochrom, Cambridge UK). GSSG and total GSH amounts were calculated by comparing sample values against the standard curve.

### Statistics

Data obtained from fibers from muscles subjected to a given test condition were statistically compared to the matching data obtained in fibers from the contralateral muscles (i.e. the relevant control), using Student's unpaired one- or two-sided *t*-test as appropriate with the probability (*P*) level set at 0.05. In the first set of both the *in vitro* and *in vivo* experiments, fibers from stimulated muscles (STIM) were compared with those from matching contralateral rested control muscles (CC). In a second set of the *in vitro* experiments, fibers from stimulated muscles were compared with fibers taken from the matching contralateral muscles which also had been stimulated but then allowed to recover for 45 min in the oxygenated bath solution before obtaining skinning fibers (STIM+REC). In a subset of the first set of experiments, skinned fibers obtained from stimulated muscles were treated with DTT *in vitro* before any activation and the results compared both to i) other skinned fibers alternately obtained from the same stimulated muscles but not treated with DTT, and to ii) skinned fibers obtained from the contralateral rested muscles similarly treated or not treated with DTT. In many experiments the force-pCa relationship in a given fiber was also re-determined after the fiber had been treated with DTT for 10 min, and in these cases the effect of DTT treatment on maximum force, pCa_50_ and *h*, was assesed by paired comparison of the relevant value in the same fiber before and after treatment, using Student's paired *t-test*.

## Results

### Whole muscle force responses

**Figure 1:**
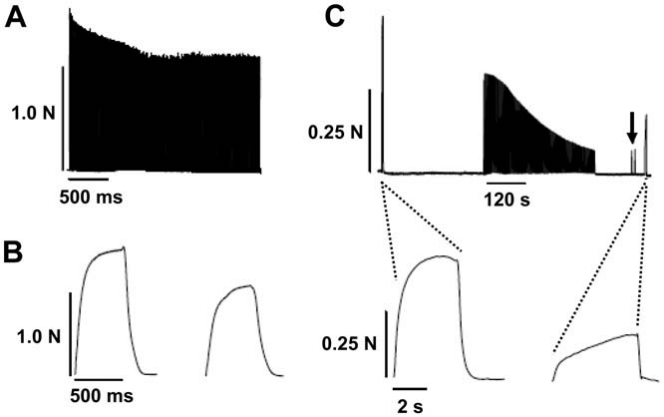
Tetanic force recordings of whole SOL muscles stimulated *in vivo* or *in vitro*. In both A & B, the main protocol involved stimulating the muscle repeatedly for 0.5 s at 50 Hz every 2 s. *A*: Tetanic force responses in a SOL muscle stimulated *in vivo* at 37°C. Peak force during repeated tetani declined only to ∼70% of initial and then remained approximately constant for the 10 min of stimulation. *B*: Force traces on expanded time scale of first and last tetani in panel A, showing that force reached close to its maximum within the 0.5 s train at 50 Hz. *C*: In a SOL muscle stimulated *in vitro* at 31°C, peak tetanic force to a 0.5 s train became progressively smaller over the stimulation protocol, dropping to <30% of the initial level after ∼6 min before stimulation was ceased. Two separate test stimuli (0.5 s trains at 50 Hz) applied 2 min later (indicated by arrow) showed there was little or no recovery of force in that time. Maximum tetanic force to 50 Hz stimulation, ascertained with a train long enough for force to plateau, was examined both before and after the stimulation protocol (traces shown on expanded time scale underneath) and displayed reduction to <40% of initial.

Before the properties of the contractile apparatus in individual fibers can be discussed, it is first necessary to consider changes to whole muscle force occurring with the stimulation protocol. In the SOL muscles stimulated *in vivo* at 37°C (e.g. Fig. 1A), peak force to 0.5 s trains of 50 Hz stimulation repeated every 2 s became smaller, declining to ∼60 to 80% of the initial level (mean 70±4%, n = 5) after 10 min of stimulation. As seen in Figure 1B (left panel), tetanic force reached close to a plateau level during the first 0.5 s tetanus, and also still did so even during the last tetanus in the 10 min protocol (Fig. 1B right panel).

In contrast to the above *in vivo* experimental findings, in the muscles stimulated *in vitro* at 31°C, the peak force reached during the first of the 0.5 s trains of 50 Hz stimulation was only 57±3% (n = 14) of the maximum force achievable with longer 50 Hz trains (e.g. Fig. 1C top). Furthermore, when such 0.5 s trains of 50 Hz stimulation were applied repeatedly every 2 s, the peak force to successive tetani dropped much faster than in the *in vivo* stimulated muscles, decreasing to <40% of the initial level within ∼2 to 6 min in SOL muscle (mean 3.1±0.6 min, n = 14, e.g. Fig. 1C). The decline in peak force to successive tetani in EDL muscle was even faster than in SOL muscle stimulated *in vitro* (mean 1.3±0.2 min, for EDL muscles, n = 3). In the ten cases examined, the force response failed to show any appreciable recovery over a subsequent two minutes (e.g. Fig. 1C), with the peak force to 0.5 s 50 Hz test stimuli being no larger than that of the last of the 0.5 s tetani in the repeated stimulation protocol (mean of peak force to last tetanus and subsequent 2 min test tetanus respectively being 22±2% and 21±4% of that elicited in the first tetanus; no significant difference, two-sided paired *t*-test). Following the repeated stimulation the maximum force achievable with a prolonged train of 50 Hz stimulation was 37±7% (n = 8) of that seen at the beginning of the experiment for SOL muscle and 18±2% (n = 3) for EDL muscle (e.g. compare right- and left-most tetanic responses in Fig. 1C for SOL muscle), which shows that maximum tetanic force to 50 Hz stimulation is reduced by the repeated stimulation to a similar extent to that seen with the briefer tetani during the stimulation protocol itself.

It is also worth noting that the absolute force elicited by the whole SOL muscles stimulated *in vitro* was far less than when they were stimulated *in vivo* (compare Fig. 1A with Fig. 1B). This phenomenon has also been shown in a study on rat extraocular muscle [41] and is most likely due both to the temperature difference (37°C vs 31°C) and the build up of extracellular K^+^ in deep regions of the *in vitro* muscle, leading to chronic depolaraization of many fibers there.

### Skinned fiber force-[Ca^2+^] characteristics

**Figure 2:**
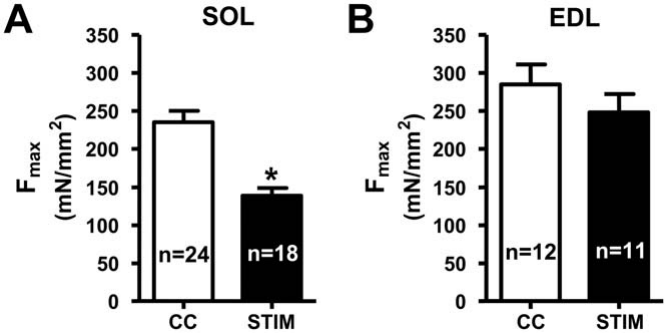
F_max_ in skinned fibers from *in vitro* stimulated and non-stimulated SOL and EDL muscles. *A*: Mean (+s.e.m) maximum specific Ca^2+^-activated force (‘F_max_’, expressed as mN/mm^2^) in skinned SOL fibers; number of fibers shown in bars. ‘STIM’ fibers from superficial regions of muscles stimulated *in vitro* as in Fig. 1C showed significantly lower F_max_ than superficial fibers from the non-stimulated contralateral control muscles (CC fibers, *P*<0.05). Data obtained from pairs of contralateral muscles from 7 rats (6 Long Evans hooded and 1 Wistar). *B*: F_max_ in superficial fibers from EDL muscles stimulated *in vitro* and matching control fibers (from 3 Long Evans hooded rats). ‘*’ denotes significantly smaller than CC fibers (*P*<0.05).

The properties of the contractile apparatus in slow-twitch fibers from SOL muscles and fast-twitch fibers from EDL muscles were determined by mechanically skinning individual fibers and examining their activation by Ca^2+^ under uniform cytoplasmic conditions (see *Materials and Methods*). F_max_ in fibers dissected from superficial regions of the *in vitro*-stimulated SOL muscles (STIM fibers, n = 18) was found to be only 59±4% of that in fibers from the non-stimulated contralateral (control) muscles (CC fibers, n = 24) (Fig. 2A). There was no significant difference in the level of reduction in F_max_ observed in the two strains of rat examined (Long Evans hooded: mean 62±13% in STIM fibers (n = 11) relative to matching CC fibers (n = 12); Wistar: 51±8% in STIM fibers (n = 7) relative to CC fibers (n = 7)) and consequently the data from the two strains were pooled together in Fig. 2A and Table 1. The decrease in F_max_ in superficial fibers from stimulated EDL muscles was substantially smaller (∼13%), and did not reach the significance level (Fig. 2B).

**Figure 3:**
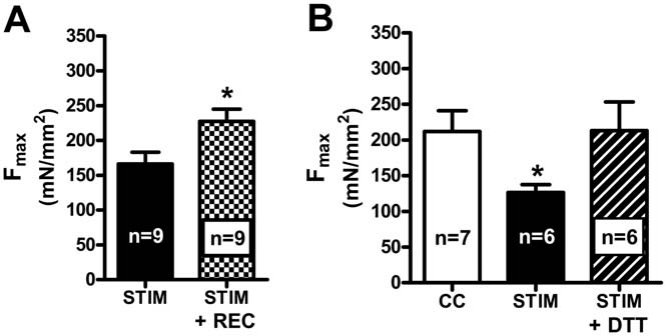
Effects of muscle recovery *in vitro* and DTT treatment of skinned fibers. *A*: Mean F_max_ (+s.e.m) in skinned fibers from SOL muscle stimulated *in vitro* (STIM, black bar) and fibers from the contralateral muscles similarly stimulated but allowed 45 min recovery in bath (STIM+REC, checkered bar). F_max_ in fibers from STIM+REC muscle was significantly larger than in fibers from STIM muscle (denoted by *, *P*<0.05). (Data from contralateral muscle pairs of 2 rats). *B*: F_max_ in skinned fibers from stimulated SOL muscles and non-stimulated contralateral muscles (CC, open bar). Alternate fibers from the stimulated muscle were examined without further treatment (STIM, n = 6, black bar) or following treatment with 10 mM DTT (10 min) prior to the first activation (STIM+DTT, n = 6, dashed bar). (Data from 2 rats). CC and STIM fibers are a subset of the data shown in Figure 2A. ‘*’ denotes significantly smaller than CC (*P*<0.05).

Fibers obtained from stimulated SOL muscles allowed to recover for 45 min in the *in vitro* bath (STIM+REC fibers) showed little if any apparent decrease in F_max_, in contrast to those fibers obtained from the contralateral muscles that were similarly stimulated but not allowed to recover (Fig. 3A). F_max_ in the STIM+REC fibers was not significantly different from that in CC fibers (compare black bar in Fig. 3A with white bar in Fig. 2A), though this comparison is made between fibers from unpaired muscles rather than between fibers obtained solely from the matching contralateral muscles. F_max_ in the STIM fibers was on average 73±11% of that in the contralateral STIM+REC fibers, which was not significantly different from the reduction observed in STIM fibers compared to CC fibers across the study (Fig. 2A).

### DTT reverses the reduction in F_max_ in skinned fibers

DTT is a potent reducing agent which is known to reverse oxidative changes to cysteine residues [42]. After the fibers had been dissected from the whole muscle and mounted onto the force transducer, they were treated with DTT for 10 min before their first Ca^2+^-activation sequence (denoted as STIM+DTT fibers) displayed significantly higher F_max_ than untreated STIM fibers obtained alternately from the same muscles (Fig. 3B), and furthermore the maximum force level in these STIM+DTT fibers was seemingly no different from that in CC fibers from the matching non-stimulated contralateral muscles (Fig. 3B). In contrast, in STIM fibers that had been activated with Ca^2+^ before being treated with DTT, subsequent DTT treatment produced no recovery of force (maximum force 167±5 mN/mm^2^ before and 159±5 mN/mm^2^ after DTT, n = 14). In CC fibers similar DTT treatment had no effect on F_max_ irrespective of whether it was applied before or after the skinned fiber underwent its first Ca^2+^-activation sequence. The fact that DTT treatment reversed the decrease in F_max_ in STIM fibers (provided it was applied before any activation) indicates that the decrease in F_max_ in the superficial fibers was due to oxidation of cysteine residues [42]. The fact that DTT treatment could not reverse the oxidative changes if the skinned fiber had been already activated is very similar to findings in fibers from heated muscles [29] (see Discussion).

### Determination of GSH: GSSG ratio in superficial fiber bundles

**Figure 4:**
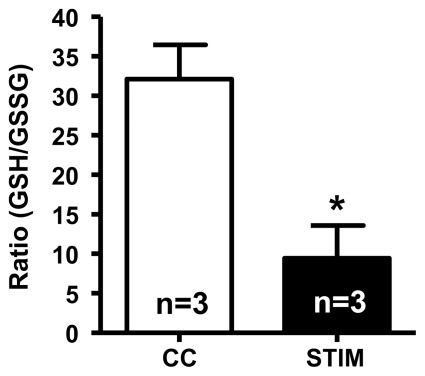
GSH∶GSSG ratio decreases in superficial fibers during *in vitro* stimulation. GSH∶GSSG ratio measured in bundles of superficial fibers from stimulated SOL muscles (STIM) and the non-stimulated contralateral control muscles (CC) from 3 rats. ‘*’ denotes STIM significantly different from CC (two-tailed paired Student's *t*-test).

The cytoplasm levels of reduced glutathione (GSH) and oxidized glutathione (GSSG) were determined in superficial fiber bundles from stimulated and control SOL muscles of 3 rats. The mean amount of GSH was 1.6±0.2 and 1.5±0.2 mmol/kg muscle in the control and stimulated fibers respectively, and the corresponding GSSG amounts were 0.06±0.01 and 0.22±0.08 mmol/kg respectively. Thus, the ratio of GSH∶GSSG was decreased ∼3 fold in the STIM fibers (mean ratio ∼28.3 versus ∼9.5 in CC fibers) (Fig. 4), demonstrating that the cytoplasm of the superficial fibers had indeed become oxidized in muscle stimulated *in vitro*.

### Tempol and L-NAME help prevent the decline in F_max_ in fibers from SOL muscle stimulated in vitro

**Figure 5:**
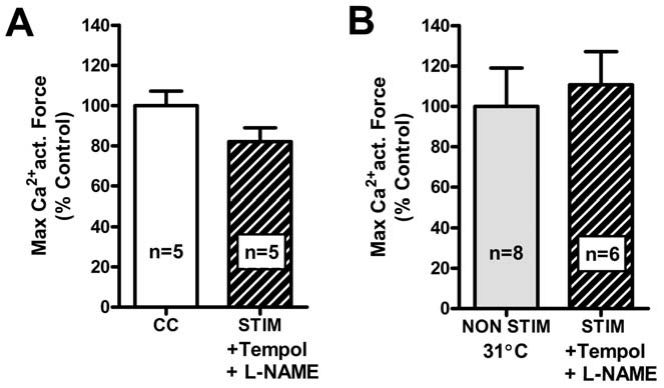
Tempol and L-NAME help prevent the decrease in F_max_. *A*: Mean relative F_max_ (+s.e.m) in fibers from non-stimulated contralateral SOL muscle (‘CC’, open bar) and SOL muscle stimulated *in vitro* in presence of 1 mM Tempol and 3 mM L-NAME (dashed bar) (all values expressed as a percentage of average of that in CC fibers recorded in same experiment). No significant difference in F_max_ between the two cases. *B*: Similar mean F_max_ data for fibers from muscles stimulated with Tempol and L-NAME present (dashed bar) and fibers from the contralateral muscles bubbled with 95% O_2_ at 31°C for same period (gray bar). No significant difference in F_max_ between the two cases.

After ascertaining that the superficial fibers were oxidized during *in vitro* stimulation, we sought to determine the oxidant(s) possibly responsible for the large reduction in F_max_ observed in the SOL fibers. The co-presence of Tempol (a superoxide dismutase mimetic) and L-NAME (a NO^•^ scavenger) during the fatigue stimulation was used to simultaneously prevent the accumulation of both NO^•^ and superoxide (O_2_^•−^), which spontaneously combine to form peroxynitrite (ONOO^−^), a potent oxidant and known to cause large reductions in F_max_ in SOL fibers [18], making it a possible candidate for the reductions in F_max_ observed here in fibers from SOL muscles stimulated *in vitro*. F_max_ in fibers dissected from these STIM+Tempol+L-NAME muscles was not significantly different (81.2±7.4%, n = 5) from that in the contralateral control fibers (Fig. 5A), and was significantly greater than in fibers from similarly stimulated muscles without Tempol and L-NAME (58.5±3.8% of matching CC fibers, n = 18, P<0.01). It was further shown that F_max_ in the fibers from muscles stimulated with Tempol and L-NAME present was not significantly different from that in fibers of the contralateral muscles bubbled with O_2_ at 31°C for the same period (Fig. 5B). The co-presence of Tempol and L-NAME had little if any effect on the whole muscle tetanic force recorded during the fatiguing stimulation (mean peak force reached during first of the 0.5 s trains of 50 Hz stimulation was 61±11% of that reached with longer 50 Hz train, and mean time to <40% initial tetanic force upon repeated stimulation was 3.2±1.6 min (n = 3 SOL muscles)).

### Unchanged Ca^2+^-sensitivity in fibers from muscles stimulated in vitro

**Figure 6:**
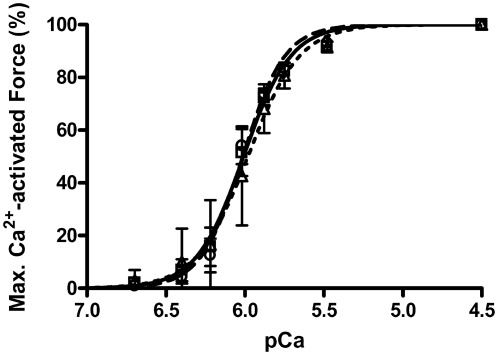
Force-pCa relationships for skinned fibers from SOL muscle stimulated *in vitro*. Average force-pCa relationships for subset of fibers used in Figures 2 and 3B; data for fibers from contralateral muscles examined in same experiment. For clarity of this summary illustration, the average of the relative force at each [Ca^2+^] was calculated across all fibers for the given condition, and a Hill curve fitted to these average force-pCa data, rather than to data for each fiber individually as in Table 1 (see note in Methods). Filled squares and solid lines: STIM (n = 6); open circles and interrupted line: CC fibers (n = 7); filled triangles and dotted line: fibers from stimulated muscles treated with DTT prior to activation: STIM+DTT (n = 6). See Table 1 for details of characteristics of Hill curves fitted to fibers individually. No differences were apparent in the force-pCa curves.

In contrast to the change in F_max_, Ca^2+^-sensitivity of the contractile apparatus of fibers was not significantly altered by the *in vitro* stimulation procedure (Fig. 6). The force-pCa relationship was determined individually for each fiber and condition (see *Materials and Methods*). Skinned fibers from both SOL and EDL muscles stimulated *in vitro* displayed no difference in force-pCa characteristics compared to fibers taken from the non-stimulated contralateral muscles. Furthermore, the force-pCa characteristics were also unaltered in fibers from the SOL muscles allowed to recover *in vitro* and in SOL skinned fibers treated with DTT (Table 1), or with and without the co-presence of Tempol and L-NAME (pCa_50_ 5.93±0.03, *h* 4.1±0.7, n = 11 compared to CC fibers pCa_50_ 5.97±0.03, *h* 4.1±0.8, n = 18; pCa50 and *h* not significantly different, *P*>0.05).

### Muscles stimulated in vivo

**Figure 7:**
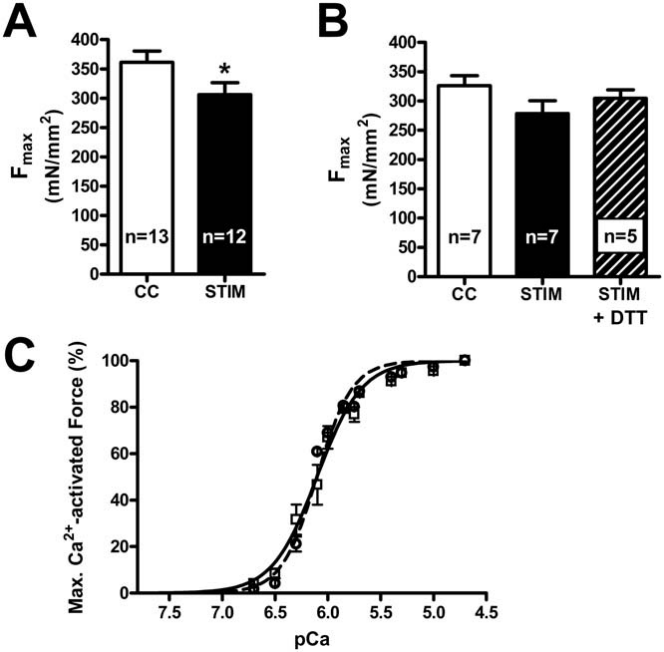
F_max_ in skinned fibers from muscle stimulated *in vivo*. *A*: Mean F_max_ (+s.e.m.) in skinned fibers from muscles stimulated *in vivo* (STIM) or from non-stimulated contralateral muscles (CC). F_max_ in STIM fibers significantly lower than in CC (fibers from 5 rats, *P*<0.05). *B*: F_max_ in skinned fibers from muscles stimulated in vivo or from contralateral controls (CC, open bar). Alternate fibers from the stimulated muscles were examined i) without additional treatment (STIM, filled bar) or ii) after treatment with DTT (10 mM, 10 min) prior to being activated (STIM+DTT, 5, dashed bar). No statistically significant differences were found (*P*>0.05). *C*: Hill curves fit to averaged force data at each pCa for all fibers for given treatment. Filled squares and solid line: fibers from stimulated muscles (n = 12); open circles and dashed line: fibers from resting contralateral muscles (n = 13). Error bars depict s.e.m. See Table 2 for details of characteristics of Hill curves fitted to fibers individually. No differences were evident.

In contrast to the *in vitro* stimulated muscle fibers, skinned fibers taken from muscles stimulated *in vivo* showed only a comparatively small depression of F_max_ (∼15% reduction, i.e. 85±5% of mean CC level) (Fig. 7A), even though the *in vivo* muscles were stimulated with the same protocol as the *in vitro* muscles for ∼two-fold longer period (10 min rather than ∼5 min, see Fig. 1) and at a temperature of 37°C compared to 31°C. Ca^2+^-sensitivity of the contractile apparatus was not significantly altered in these fibers (see Fig. 7C, mean values for pCa_50_ and *h* in Table 2). In a subset of the *in vivo* experiments shown in Fig. 7A, alternate fibers from STIM muscles were either treated or not treated with DTT before being first activated; although the mean specific force in these STIM+DTT fibers was nominally higher than in the matching STIM fibers (Fig. 7B), the difference was not statistically significant.

### Consideration of possible dissection time issue

In order to investigate whether the properties of the fibers might have altered or recovered during the period before skinning when the muscles were kept cool on ice, values of the maximum specific force measured in each fiber were plotted against the time elapsed between placing the whole muscle in the oil on the ice and mounting the given skinned fiber (data not shown). F_max_ remained unchanged over the period before fiber dissection in the cases of both the STIM and CC fibers, as indicated by the lack of correlation between F_max_ and time in dish (r^2^ = 0.0918 and 0.0046 for CC and STIM fibers respectively). Similarly, there was no evidence of any change in Ca^2+^-sensitivity of the fibers over the period before dissection (not shown).

### Consideration of effect of possible fiber swelling on F_max_

As F_max_ was normalized to fiber cross-sectional area, we considered whether the values found could have been influenced by fiber swelling with stimulation. It was apparent that this could not account for the large difference in specific force found. Firstly, there was no evidence for any large alteration in cell cross-sectional area in the stimulated fibers, as average cross-sectional area of the dissected fibers was not significantly different between groups, and total muscle weight (after removing excess buffer solution by briefly blotting the muscle) was little increased following muscle stimulation (<5%, n = 7). Furthermore, fiber absolute maximum force followed the same trend as the values normalised by fiber cross-sectional area. Finally, the reduction in specific force was not seen if skinned fibers from stimulated muscles were treated with DTT (see earlier).

## Discussion

This study found that intense fatiguing stimulation of whole SOL muscle *in vitro* in the presence of ample oxygenation resulted in a marked ROS-mediated reduction in the F_max_ in superficial fibers, without any change to Ca^2+^-sensitivity. It was possible to assess the properties of the contractile apparatus without confounding effects arising from the acute cytoplasmic changes that occur with muscle activity [25] by examining skinned muscle fibers under set cytoplasmic conditions. It was found that F_max_ was restored to the control level if the stimulated fibers were allowed to recover for 45 min whilst still intact, or if the skinned fibers were treated with DTT (a potent reducing agent) prior to activation, the latter indicative that the reduction in F_max_ was due to an oxidation reaction. The GSH∶GSSG ratio in the cytoplasm of superficial fibers of stimulated muscles was found to be ∼3 fold lower than in non-stimulated control muscles, consistent with exposure to elevated levels of oxidants. Furthermore, the presence of Tempol and L-NAME during *in vitro* stimulation prevented the reduction to F_max_, indicative that the deleterious effects were due to superoxide and/or nitric oxide, or related compounds.

### Possible role of peroxynitrite in decreasing F_max_ in stimulated muscle fibers

Various ROS evidently cause different characteristic changes in the properties of the contractile apparatus. For example, in rat skinned SOL and EDL fibers H_2_O_2_ is relatively ineffective, with a 5 min exposure to 10 mM H_2_O_2_ causing only a small decrease in the Hill coefficient with little or no effect of F_max_ [12] (similar to findings in diaphragm [11]), whereas OH^•^ causes concomitant decrease in both Ca^2+^-sensitivity and F_max_ [13]. Nitric oxide (NO^•^) donors, GSNO or SNAP, on the other hand, cause substantial Ca^2+^-sensitivity changes in fast-twitch (EDL) fibers but have little effect on F_max_ except at very high concentration [17], [19]. In contrast to the above oxidants, peroxynitrite causes a marked decrease in F_max_ in skinned SOL fibers with little change in Ca^2+^-sensitivity, and has relatively less effect in EDL fibers [18], strikingly like that found here in the stimulated fibers (Figs. 2 & 6). Peroxynitrite also decreases F_max_ in diaphragm fibers without changing Ca^2+^-sensitivity [35]. Thus, it seems quite plausible that the effects seen here were due to action(s) of peroxynitrite generated within the superficial fibers of the stimulated muscles. Superoxide and NO^•^ are both generated in active muscles, particularly with supra-physiological oxygen tensions [27], [34], and readily react to form peroxynitrite [31], [32], [33], [34]. The findings here are also very similar to those observed in SOL fiber bundles made hypoxic for 30 min, where F_max_ was reduced without change in Ca^2+^-sensitivity, and with the effect being prevented by the NOS inhibitor L-NMMA, consistent with it being due to peroxynitrite. [30]. Furthermore, peroxynitrite has been shown to inhibit actin-activated ATPase activity of myosin heads [43], which would seemingly account for the reduction in F_max_ seen here. It is possible nevertheless that the effects were due at least in part to superoxide itself, as it also has been found to decrease F_max_ without change in Ca^2+^-sensitivity in cardiac muscle cells [44] and to decrease F_max_ in diaphragm fibers (with unknown effects on Ca^2+^-sensitivity) [11].

Interestingly, it was found here that slow-twitch SOL fibers showed greater ROS-related dysfunction than did fast-twitch EDL fibers, despite the fact that SOL fibers have ∼5-fold more of the endogenous antioxidant GSH than do fast-twitch fibers [45]. This may reflect that SOL fibers not only are more susceptible to a given level of peroxynitrite than EDL fibers [18], but also possibly generate greater levels of peroxynitrite and related radicals when stimulated *in vitro* under these conditions due to their great oxidative metabolism.

### Reversibilty with recovery and with DTT treatment

The finding that F_max_ restored to control levels if fibers were left intact for a 45 min period after stimulation (Fig. 3) is also very similar to the restoration of F_max_ after recovery from both hypoxia [30] and heat treatment [29]. Presumably, such a recovery period allows reducing factors present endogenously in the normal cytoplasm (e.g. glutathione and glutathione reductase) to reverse the oxidative changes to the contractile proteins. Such factors, however, are lost when muscle fibers are skinned and bathed in the standard intracellular solution, and consequently any oxidative changes can then only be reversed by application of an exogenous reducing agent.

Interestingly, the ability of DTT to reverse the reduction in F_max_ in fibers from stimulated muscle was entirely dependent on whether or not the skinned fiber had been activated whilst still oxidized; if a STIM fiber was activated in the Ca^2+^ solutions and then DTT applied, there was little or no reversal of F_max_ (see Results), but if the DTT was applied before activation, F_max_ recovered to the control level (Fig. 3B). Exactly the same type of effect was found previously when attempting to reverse the F_max_ decrease in heat-treated muscles [29] and also in fibers directly treated with OH^•^ [13] and peroxynitrite [18]. This activation-dependent interference with the ability of DTT to reverse the F_max_ decrease readily explains why similar effects of peroxynitrite on F_max_ in cardiomyocytes were poorly reversible with DTT and other reducing agents [46], and these agents were only applied after first activating the cells to show the decrease in F_max._ It might similarly explain why DTT failed to reverse the putative ROS-mediated decrease in F_max_ seen in endotoxin-treated diaphragm fibers, which also were activated before DTT was applied [47]. The basis of the phenomenon however remains unclear. One possibility is that it results from unfolding of the myosin heads during the relatively prolonged Ca^2+^-activation of the contractile apparatus used in the skinned fiber experiments, as such unfolding of the myosin head was found to readily occur following peroxynitrite-induced oxidation [43]. Alternatively, activation of the contractile apparatus might expose or bring into close proximity particular oxidised and reduced cysteine residues, which leads to subsequent irreversible structural changes.

### Potential ROS-mediated problems in muscles stimulated in vitro and in situ

The results presented here demonstrate that ROS-mediated dysfunction of the contractile proteins readily occurs in *in vitro* muscle preparations not only with hypoxia [30] and heat-treatment [28], [29], but even in well-oxygenated superficial fibers in stimulated muscles. The common factor in all these cases of course is the generation of large amounts of ROS/RNS in each of these situations [23], [24], [26], [27], [36]. Thus, the findings here highlight a potential difficulty in experiments investigating mechanisms of muscle function and fatigue that involve applying intense stimulation to *in vitro* muscle preparations, as measurements of the rate or extent of force decline could be unduly affected by ROS-mediated dysfunction of the contractile apparatus and perhaps also other key processes. For example, we note that the *in vitro* soleus muscle preparation and protocol used to infer that aberrant ryanodine receptor function underlies skeletal muscle dysfunction in a rat cardiac heart failure (CHF) model [48], was almost exactly the same as that examined here, leaving some question as to whether ROS-mediated dysfunction of the contractile proteins may have contributed to the poorer force responses observed in the CHF muscles.

It is interesting to note that *in vitro* muscle preparations consisting of just a single or a few isolated fibers and perfused with high O_2_ seem to maintain force development to repeated stimulation better than whole muscle preparations [49] even at high temperatures [50], suggesting that any deleterious ROS effects are much less in such thin preparations. Owing to the very thin nature of those preparations and the high diffusibility across the surface membrane of NO^•^ and ROS such as H_2_O_2_, it seems likely that there would be much less accumulation of ROS and NO^•^ within the fibers in such preparations compared to those in whole muscles stimulated *in vitro*.

Importantly, there was reduction in F_max_, without change in Ca^2+^-sensitivity, even in the fibers from SOL muscles stimulated *in situ* with their blood supply intact (Fig. 7), with the effects being smaller after DTT treatment, although the latter did not reach the significance level. This finding in the *in situ* stimulated muscles however cannot be taken to imply that similar dysfunction necessarily also occurs in the muscles of exercising animals and humans. For one thing the *in situ* stimulated SOL muscles here were subjected to relatively intense and prolonged stimulation (50 Hz for 0.5 sec every 2 sec continuously for 10 min), whereas the firing rate of neural stimulation to motor units *in vivo* is set at the lowest level for optimal muscle response, with the rate declining with continued stimulation and fatigue [51], [52]. Consequently, the rate of generation of ROS and RNS may have been artificially high in the *in situ* experiments here. Secondly, as the experimental animals were anesthetized in the *in situ* stimulation experiments, there would have been little or none of the normal upregulation of cardio-respiratory system that occurs during exercise, nor movement related increases in muscle perfusion, and hence the accumulation of ROS and RNS were likely higher in the muscles here compared to that in exercising animals.

### ROS-related reduction in F_max_ and disease

The ROS-mediated dysfunction seen here in fibers from the *in vitro* stimulated muscle, as well as in many other *in vitro* preparations [28], [29], [30], and likely also in the *in situ* stimulated muscle, are strikingly similar to those observed in many pathological conditions, which highlights how readily excess ROS/RNS generation or accumulation can lead to muscle progressing from a normal to a pathological state. For example, inflammation has been found to lead to TNFα-mediated increase in ROS/RNS in diaphragm fibers, resulting in a reduction in F_max_ with no change in Ca^2+^-sensitivity [3], [9]. Similar effects of limb and diaphragm muscle are also seen with sepsis and endotoxin application [47], [53], and in CHF [8], [54], [55].

An interesting related issue that still needs to be resolved is whether the dysfunction in F_max_ occurring in these various pathological conditions can be readily reversed by reducing treatments, such as application of DTT or similar agents. As mentioned earlier, it was found that DTT application did not reverse the decrease in F_max_ seen in endotoxin-treated muscle fibers [47], but this was only investigated after the skinned fibers used to assess the force response had been already activated. It is important to know for the various pathological cases whether F_max_ can be restored by DTT applied either to skinned fibers that had not been already activated or to preparations of intact muscle fibers. This would indicate whether the contractile apparatus changes in the pathological states are fully comparable with those seen in fibers after acute oxidizing treatments, or whether instead the pathological changes are more irreversible, as could happen if the critical sites (presumable cysteine residues) have been subjected to particularly prolonged or intense oxidative stress, leading to progression from reversible cysteine oxidation states (i.e. sulfenation, nitrosylation, glutathionylation or disulfide links) to poorly reversible or irreversible states (e.g. sulfination or sulfonation) [56], [57].

### Concluding remarks

In conclusion, this study shows that despite ample oxygenation, ROS-mediated dysfunction of the contractile apparatus readily occurs even in superficial fibers of SOL muscles stimulated *in vitro*, and to a lesser extent even in muscles stimulated *in situ* with an intact blood supply. The large decrease in F_max_ showed all the hallmarks of peroxynitrite-induced oxidation. The ROS/RNS-mediated changes to the contractile apparatus observed here closely mimic the muscle dysfunction observed in many conditions, such as vascular disease, ischaemia-reperfusion injury, sepsis, inflammatory conditions, heart failure, stroke and rheumatoid arthritis, and highlights the ease of progression from normal to pathological states. Thus, deriving an understanding of the mechanisms involved in ROS-related contractile dysfunction in normal healthy muscle could help provide valuable insight into an array of various diseases and pathophysiological conditions.
